# A New Ligature

**Published:** 1878-08

**Authors:** Edward C. Huse

**Affiliations:** Rockford, Ills.


*A New Ligature.*

The carbolized cat-gut ligatures which Prof. Lister has introduced to the profession, are of great advantage over the time-honored silken ones, or those formed from silver wire. Torsion, acupuncture and the various devices in vogue from time to time, have each had their friends and enemies, their advantages and disadvantages. The principal danger in using any and all ligatures especially upon vessels of the first magnitude, such as the subclavian, the carotid, or the iliac, is secondary hæmorrhage. Septicæmia is not likely to occur after using carbolized ligatures, yet the chief objection to them is that the material of which they are composed is organic. Hence, like all organic substances, they are liable to be either imperfect in structure and break, to be imperfectly carbolized and hence rot, or to produce irritant and septic effects at the point of application. It is not easy to see how the multiple conditions on which their absorption depends, can, in every instance, be complied with, especially outside the walls of a hospital. At best and in any event, the life of a patient, where one of these ligatures has been used, hangs upon little more than a thread and often far less than that. A ligature, to be perfect of its kind, should possess the elements of strength, ease of application, readiness of absorption and simplicity. It should not depend for its advantages upon a material perishable in its nature under *ordinary* circumstances, neither should its perishability and consequent absorption on which its whole value depends, be left to accident or chance.

There is but one substance in nature which entirely meets and fulfills the requisites of a perfect ligature. This substance the writer has carefully tried in several instances; and begs leave to submit his plan unhesitatingly to the profession.

If a piece of magnesium wire be lighted, as it readily is, in the flame of a lamp, and allowed to burn, the process of oxidation which ensues upon its combustion, converts it into ordinary magnesia. The ash which burning magnesium forms, is ordinary magnesia, plain and simple. It is thus innocuous and readily absorbed.

Its action upon the animal economy is absolutely harmless to all textures everywhere. When an artery or vein is tied with a loop of magnesium wire, this process of oxidation goes on more slowly in the presence of animal heat and moisture, yet none the less surely, none the less completely than when it is burned up in the way above cited.

As fast as it is oxidized it is absorbed in the form of magnesia, whose effect, if anything, would be, from its alkaline character, to prevent rather than excite irritation. The length of time required for its absorption depends of course upon the size of the wire used. It is a matter of no importance comparatively, whether it be absorbed in ten days or one hundred if we know *it will be absorbed* beyond a peradventure in all instances. Thus far I have used it but three times, once upon the radial artery and twice in the operation for varicocele. It has seemed to me that this ligature will be of a special value in ovariotomy, where it is desirable to tie the vessels of the pedicle and return it into the abdomen, and in operations for hemorrhoids. It can be used everywhere under all conceivable circumstances and contingencies; it will never break; it is always ready; it cannot untwist like cat-gut or silk; it can neither slip, become stiff or rotten; it can never provoke irritation, absorb moisture, disappoint or cause anxiety, but will always act, "*tuto cito et jucunde.*"

It seems to me, finally, that it must supersede all ligatures, because it is not only better, safer, more convenient and needful, but the *only* thing necessary to overcome the whole category of objections on either one of which, lives of priceless value have in hosts of cases been dependent.

I would earnestly beg that this discovery may receive careful and unbiased attention, as I am confidently assured that it will not disappoint surgeons or patients.

EDWARD C. HUSE, M. D.

ROCKFORD, ILLS., June 17th, 1878.

